# *Jaxkineticmodel*: Neural ordinary differential equations inspired parameterization of kinetic models

**DOI:** 10.1371/journal.pcbi.1012733

**Published:** 2025-07-07

**Authors:** Paul van Lent, Olga Bunkova, Bálint Magyar, Léon Planken, Joep Schmitz, Thomas Abeel

**Affiliations:** 1 Intelligent Systems, Delft University of Technology, Delft, Zuid-Holland, Netherlands; 2 Department of Science and Research, dsm-firmenich, Delft, Zuid-Holland, Netherlands; 3 Infectious Disease and Microbiome Program, Broad Institute of MIT and Harvard, Cambridge, Massachusetts, United States of America; Inria Saclay: Inria Centre de Recherche Saclay-Ile-de-France, FRANCE

## Abstract

**Motivation:** Metabolic kinetic models are widely used to model biological systems. Despite their widespread use, it remains challenging to parameterize these Ordinary Differential Equations (ODE) for large scale kinetic models. Recent work on neural ODEs has shown the potential for modeling time-series data using neural networks, and many methodological developments in this field can similarly be applied to kinetic models.

**Results:** We have implemented a simulation and training framework for Systems Biology Markup Language (SBML) models using JAX/Diffrax, which we named *jaxkineticmodel*. JAX allows for automatic differentiation and just-in-time compilation capabilities to speed up the parameterization of kinetic models, while also allowing for hybridizing kinetic models with neural networks. We show the robust capabilities of training kinetic models using this framework on a large collection of SBML models with different degrees of prior information on parameter initialization. We furthermore showcase the training framework implementation on a complex model of glycolysis. Finally, we show an example of hybridizing kinetic model with a neural network if a reaction mechanism is unknown. These results show that our framework can be used to fit large metabolic kinetic models efficiently and provides a strong platform for modeling biological systems.

**Implementation:** Implementation of *jaxkineticmodel* is available as a Python package at https://github.com/AbeelLab/jaxkineticmodel.

## Author summary

Understanding how metabolism works from a systems perspective is important for many biotechnological applications. Metabolic kinetic models help in achieving understanding, but their construction and parametrization have proven to be complex, especially for larger metabolic networks. Recent success in the field of neural ordinary differential equations in combination with other mathematical/computational techniques may help in tackling this issue for training kinetic models. We have implemented a Python package named *jaxkineticmodel* that can be used to build, simulate, and train kinetic models, as well as compatibility with the Systems Biology Markup Language. This framework allows for efficient training of kinetic models on time-series concentration data using a neural ordinary differential equation inspired approach. We show the convergence properties on a large collection of SBML models, as well as experimental data. This shows a robust training process for models with hundreds of parameters, indicating that it can be used for large-scale kinetic model training.

## Introduction

Kinetic modeling is a useful tool for describing biological systems in a quantitative manner, with many applications in the biotechnological and medical domain [1,2]. In the biotechnological domain, the application of kinetic models includes assessing metabolic control of pathways [3], simulation of metabolic engineering scenarios [4,5], and optimizing feeding strategies on the bioprocess level [6]. Effective deployment of kinetic models for these purposes requires a representative description of the biological process in mathematical equations, as well as fitting the model parameters to available data. The encountered data in this domain is typically limited and either steady-state or dynamic in nature.

Metabolic kinetic models are described by ODEs that describe the change over time of metabolites (*m*) by a right-hand-side formula that consists of the mass balances imposed by the stoichiometric matrix (*S*) and a vector of reaction flux functions ($\vec{v}$) (Eq 1). The process of finding a model that reproduces observed data therefore consists of establishing the stoichiometric matrix [7,8], determining kinetic mechanisms [9], and parametrization of the flux functions.

$$\frac{dm(t)}{dt}=S\cdot v(t,m(t),\theta )$$ (1)

Fitting parameters to large-scale kinetic models can be challenging [10]. While biological systems operate on many different timescales, biologically relevant parameters can vary orders of magnitude [11]. Additionally, the ODEs observables might be insensitive to many of these parameters, sometimes referred to as sloppiness [12]. Furthermore, many kinetic models in systems biology have unidentifiable parameters, which complicates the fitting process even more [13]. Finally, many biological systems are known to be stiff [14], which leads to problems when numerically solving the ODEs [15]. These challenges complicate the use of standard parameter estimation methods.

Several parameter estimation methods have been proposed and implemented in publicly available software packages to tackle this difficult task (see S1 text). Some methods focus specifically on fitting steady-state fluxomics and metabolomics data; through gradient-based optimization [16,17], sampling-based approaches [18,19], generative modeling [20,21], or Bayesian approaches [22]. Other toolboxes provide more general purpose parameter inference methods for metabolic modeling, such as pyPESTO [23]. pyPESTO provides an interface to many different optimization methods: local versus global, gradient-free versus gradient-based optimizers. A particular efficient method that is integrated in pyPESTO are the multi-start gradient-based methods, which uses AMICI for efficient sensitivity computation [24]. These multi-start, gradient-based methods have shown to perform well on large-scale fitting problems [25,26].

Recently, Neural ODEs were introduced [27] and applied to modeling time-series concentration data [28], as well as in other applications in the biological domain [29]. The idea behind a neural ODE is to replace the right-hand-side of Eq 1 by a neural network and then use a numerical solver to predict a time-series. The neural network is then trained using back-propagation with the adjoint state method; a very efficient method for estimating gradients. These methods are efficient compared to forward gradient computation, as they do not scale with the number of model parameters: a feature that is important in neural networks or large kinetic models (see Fig A and Table C in S1 text) [20,27]. Even though neural ODEs lack the necessary mechanistic structure required in biotechnological/medical applications, many of the techniques for training, such as memory efficient adjoint gradient computation for time-series data, can similarly be applied to metabolic kinetic models. Furthermore, hybrid approaches, where a mechanistic model is augmented with a neural network to model dynamics that are not mechanistically understood, are increasingly being studied and used [30–32].

**Fig 1:**
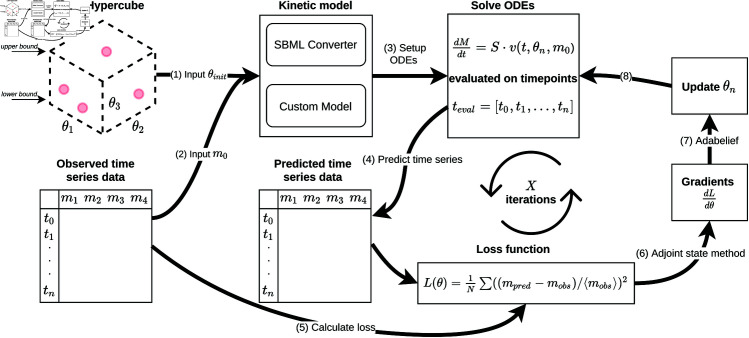
Overview of the implemented simulation tool and training framework in Diffrax. Latin Hypercube sampling is used to initialize parameters given a lower- and upper bound value (1). The initial conditions are retrieved from the observed dataset (2) and the ODEs are set up based on an imported SBML model, or a self-implemented model (3). After predicting a time-series dataset given the initial guess (4), the mean-centered loss is calculated (5). The gradients that are calculated through the adjoint state method (6) are then used to update parameters using AdaBelief (7)[40]. This process is repeated for *X* steps or until convergence (8). Implementation details on specific aspects are discussed in *Design and Implementation*.

In this work, we have implemented a *JAX*-based training and model building framework [33] for systems biology models, which we named *jaxkineticmodel* (Fig 1). *JAX* has just-in-time compilation, automatic differentiation, and parallelization capabilities, which paves the way to large-scale kinetic model training. Even though similar features have been implemented in packages for the *Julia* programming language [75], having Python packages for systems biology purposes is valuable due to its widespread use and support [67]. Furthermore, while a previous python package *SBMLtoODEjax* was developed that provides an interface between SBML and *JAX* [67], several unique features are implemented. First, *jaxkineticmodel* supports a larger set of SBML models (see Table D in S1 text), is compatible with numerical solvers from *Diffrax* [34], optimizers from *Optax* [35] and has the capability to integrate mechanistic and neural network components [32]. The default training framework is tailored to systems biology, with support for the Systems Biology Markup Language, as well as manual model building using predefined kinetic mechanisms.[36]. As a default setting, training is performed by gradient descent in log parameter space [11] with a stiff numerical solver [37] and a custom loss function to deal with metabolic scale differences. Gradients are calculated using an efficient adjoint state method from the *Diffrax* package [34]. To further stabilize training, we perform gradient clipping, a method that is used in stabilizing the training process of recurrent neural networks and neural ODEs [38]. We apply this implementation on a large collection of SBML models to answer questions on robustness of the training procedure in terms of convergence properties. Furthermore, we show the training of a large-scale kinetic model of glycolysis (141 parameters) to model feast/famine feeding strategy datasets [39]. Finally, we show how *jaxkineticmodel* can be used for hybrid models that have mechanistic and neural network components, an increasingly important application of universal differential equations in systems biology [30,32].

## Design and implementation

### Implementation

An overview of the training framework is shown in Fig 1. All experiments, code, and results, as well as documentation are publicly available. Internally, *jaxkineticmodel* uses *Sympy* [41], *JAX* [33], and *LibSBML* [36] for compatibility with many numerical solvers in *Diffrax* [34]. Documentation is available on Github.

#### Kinetic model setup.

We provide two options to set up the system of ODEs required prior to training. A model can be constructed by using kinetic mechanisms that have been provided in *jaxkineticmodel* (see Table A in S1 text), or by using the SBML-to-JAX converter. Kinetic models can also be exported to the SBML format after parameterization [36].

#### Scaling of species in the loss function.

Biological systems exhibit large-scale differences in metabolite concentrations, therefore resulting in mean squared error loss *J* being dominated by large absolute error, even though relative error might be small [15]. We therefore implemented as a default a mean-centered loss function (Eq 2), but other loss functions can be passed as well.

$$J(m_{pred},m_{observed})=\frac{1}{N}\sum (\frac{m_{pred}-m_{observed}}{\langle m_{observed}\rangle }{)}^{2}$$ (2)

#### Specifics of the gradient descent algorithm.

The AdaBelief optimizer was used during training [40]. Training was further stabilized by clipping the gradient global norm to prevent the exploding gradient problem often encountered in neural ODEs and recurrent neural networks[38]. This requires setting a maximum global norm hyperparameter ($\hat{g}$), which was set to $\hat{g}=4$. As previous reports show that systems biology models are better fitted in a log-transformed parameter space [11,42], we applied the log-transformation of parameters before passing it through AdaBelief. We note however that users can change the optimizer to any optimizer provided in *Optax* [35].

#### Specifics of the solver.

Simulations were performed using the Kvaerno5 stiff ODE solver, which was already implemented in *Diffrax* [37]. The relative and absolute error tolerances were 10^−8^ and 10^−11^, respectively. The initial time step *dt*_0_ was set to 10^−10^. *Jaxkineticmodel* is compatible with many numerical solvers for ODEs in *Diffrax*. For the adjoint state method, the default implementation provided by *Diffrax* is used, but users can also change this according to their preferences [34].

### Data

#### SBML models.

To rigorously test properties of *jaxkineticmodel*, we generate time-series metabolomics datasets from SBML models. The advantages of using synthetic data are that we can: 1) compare learned parameters against true parameters; and 2) test the method against a large collection of biological systems. The twenty-six models were retrieved from a previously reported set of benchmark models [11] and the Biomodels database [43]. These models are both dynamic models and steady-state models. Relevant properties of the models are summarized in Table 1. Models were simulated on a time interval *t* = *[*0,*t*_*end*_*]* with ten data points per specimen for the ground truth parameters. We chose ten observations as in many biotechnological domains, time-series data is only sparsely sampled. No noise model was used for the observations.

**Table 1:** Overview of the SBML models used in this study. The collection of models was retrieved from Biomodels [43] and a previously reported collection of SBML benchmark models [11]. The number of parameters, number of species, simulated range, and data points are reported.

Model	Parameters	Species	$t_{end}$	Data points
Garde *et al*. 2020 [44]	6	6	6	60
Smallbone *et al*. (2013) [45]	10	2	10	20
Bruno *et al*. (2016) [46]	10	6	100	60
Beer *et al*. (2014) [47]	12	4	8000	40
Patil *et al*. (2023) [48]	13	12	200	60
Palani *et al*. (2011) [49]	15	5	2500	50
Crauste *et al*. (2017) [50]	16	5	15	60
Sneyd *et al*. (2002) [51]	16	6	2	60
Becker *et al*. (2010) [52]	17	6	15	60
Brannmark *et al*. (2010) [54]	18	9	50	90
Ray *et al*. (2013) [55]	20	6	25	60
Elowitz *et al*. (2000) [56]	22	8	30	80
Fiedler *et al*. (2016) [57]	24	6	10	60
Borghans *et al*. (1997) [58]	24	3	3	30
Fujita *et al*. (2010) [59]	26	9	2000	90
Bertozzi *et al*. (2020) [60]	36	3	200	30
Hass *et al*. (2017) [61]	37	9	100	90
Raia *et al*. (2011) [62]	45	14	150	140
Smallbone *et al*. (2011) [63]	52	6	10	60
Weber *et al*. (2015) [64]	53	7	10	70
Zheng *et al*. (2012) [65]	62	15	100	150
Isensee *et al*. (2018) [66]	63	25	100	250
Chassagnole *et al*. (2002) [68]	117	36	10	360
Messiha *et al*. (2014) [69]	192	28	10	280
Mosbacher (2023) [70]	280	95	10	980

#### Feast/famine cycle and steady-state data.

Along synthetic data, we applied the parameterization framework to datasets of a feast/famine cycle that was previously reported [39]. Feast/famine experiments are a stimulus-response experiment that consists of a 20 second feeding phase, followed by 380 seconds without feed. Three datasets were available for fitting a glycolysis model.

### Evaluation of training framework

#### Gradient averaging for parameterization using multiple datasets.

To showcase the practical usability of Neural ODEs, we aim to fit multiple datasets to the glycolysis model (see S1 text). These datasets are both steady-state metabolomics for different dilution rates, as well as dynamic data in the form of a glucose pulse. In order to simultaneously fit all datasets, we calculate the gradient dJ/dθ for each individual dataset and average them before updating using AdaBelief. This is a popular method to train a global model from local datasets in federated learning [71].

### Evaluating the training process on simulated data

Three properties of the training process are tested after training: 1) the initialization success rate of parameters; 2) the convergence properties through the relative improvement over the initialization; and 3) the distance of trained parameters to the true optimum. All experiments are performed three times.

#### Initialization success percentage.

Datasets are simulated with the true parameters ($\theta_{true})$ that are specified in each SBML model. Latin hypercube sampling [72] is used to initialize 100 parameters within lower and upper bounds of the true parameter values, i.e., $\theta_{true}/X\leq \theta_{true}\leq X\theta_{true}$. To investigate the effect of priors on initialization success, we chose priors for five different values of *X*: 2, 5, 10, 50, and 100. The initialization success rate, that is, the first iteration of stochastic gradient descent that leads to an estimate of the error, is calculated as a percentage of the total number of initializations.

#### Convergence of successful parameter initializations.

For successful initializations, training is performed with 3000 iterations of stochastic gradient descent (AdaBelief) per initial parameter guess. To reduce computation time, training is stopped early when the loss function is below the loss threshold $\lambda =10^{-6}$ . To quantify convergence properties, we look at the relative improvement in the mean squared error after training with respect to the initialization loss, as well as the percentage of successful training (λ<0.001).

#### Global norm of gradients.

The magnitude of the gradients with respect to the parameters is followed during the training process using the L2 norm (Eq 3). This allows to investigate the training process in more detail for different models.

$$||\nabla J(\theta )||=\sqrt{(\frac{\partial J}{\partial \theta_{1}}{)}^{2}+(\frac{\partial J}{\partial \theta_{2}}{)}^{2}+...+(\frac{\partial J}{\partial \theta_{n}}{)}^{2}}$$ (3)

### Fitting the glycolysis model

To showcase the usefulness of the proposed framework we fit feast/famine datasets to a previously established kinetic model of glycolysis [73]. The model was reimplemented to be compatible with *JAX* to make parameters trainable [33]. The parameter initialization was done from parameters retrieved from literature. The model consists of 29 metabolites and 38 reactions, totaling 141 parameters. Twenty distinct rate laws were implemented as reusable *JAX* classes. Details on the implementation are reported in Table A in S1 text.

### Hybrid modeling example

A minimal model of metabolic oscillations in biofilms was used to showcase the usefulness of automatic differentiation capabilities in *JAX/Diffrax* [44]. Reaction names in the original model were replaced by symbolic names for conciseness ($v_{1}$-$v_{10}$). In this setup, we assume that a certain reaction and its stoichiometry are unknown, a scenario that is often encountered in real-world applications. The *i*-th reaction of the model of the fully mechanistic description (Eq 1) is then replaced by a neural network (Eq 4).

$$\frac{dm(t)}{dt}=S^{\left(-i\right)}\cdot v^{\left(-i\right)}(t,m(t),\theta )\;+NN(t,y,w,b)$$ (4)

We perform a masking experiment for every reaction in the model, with 100 time-points and a noise percentage of 0.05%. All reactions and the stoichiometry were individually masked.

## Results

### Training SBML models using *Diffrax*

In order to analyze the behavior of the parametrization using techniques from neural ODEs, an efficient and easy-to-use ODE simulation tool for systems biology models with automatic differentiation options for calculating adjoint sensitivities was required. We have implemented a *JAX*-based simulation tool and training framework that is compatible with Systems Biology Markup Language (SBML) models in *Diffrax* [33,34] (Fig 1). SBML models are the standard accepted format for saving systems biology models in a reproducible manner [36].

The training input consists of three information modes. The kinetic model is loaded from an SBML format or a manually implemented *JAX*-compatible class that allows for Just-In-Time (JIT) compiling. The observed time-series data that is used for fitting is used to get the initial conditions from *t*_0_. Finally, an initial parameter guess is required, which was obtained using Latin Hypercube Sampling [72]. Due to nonlinearities and potential non-convexity of the solution space, multiple initialization are typically required.

For the training process, the ODEs are solved for timepoints that are observed in the dataset and the loss function is calculated. Due to large differences in metabolite concentration ranges, a mean-centered loss function was used to ensure roughly equal contribution of metabolites to the mean squared error. Finally, *N* iterations of stochastic gradient descent using AdaBelief can be performed [40].

While kinetic models typically have a mechanistic structure of the right-hand-side of dm(t)/dt, similar tools that are used to train Neural ODEs (e.g., the adjoint state method) can be applied. The goal of the study performed here is to address properties on the convergence of local gradient-based methods for a large collection of systems biology models.

### Loss convergence analysis reveals robust training of systems biology models

Training kinetic models by gradient descent requires an initial guess of parameters. This requires setting a lower- and upper bound of parameters for the initialization and sampling the space using any sampling method. Due to the dependency of the loss function on numerical integration of the ODEs, not every parameter initialization might be successful. This can be attributed to either stiffness of the dynamical system or unstable behavior of the systems given that particular initial guess [15]. We therefore aim to understand how loss convergence is affected by parameter priors and model size, as well as how robust the training process is to these features by quantifying the percentage of successfully trained models given the parameter initializations.

**Fig 2:**
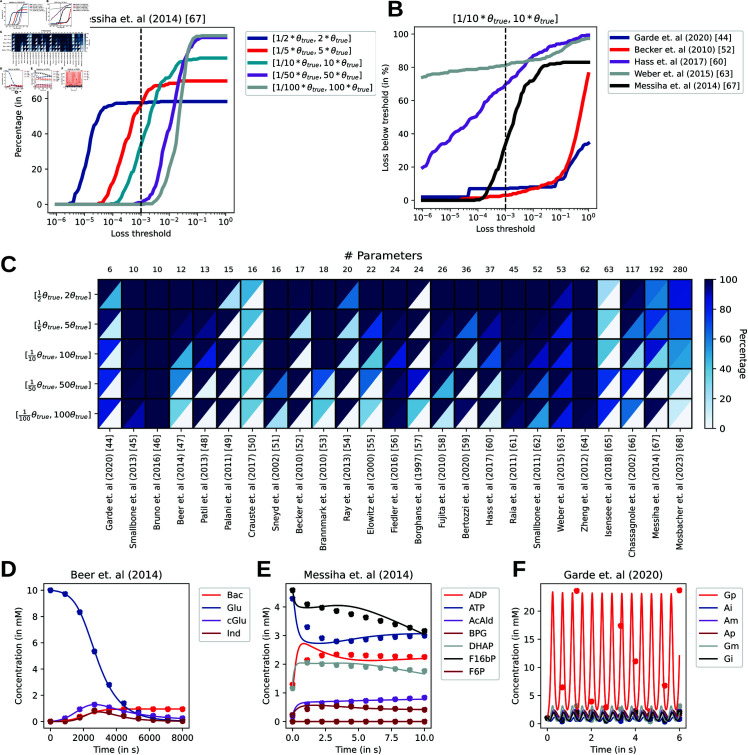
Analysis of convergence properties for a collection of SBML models. Latin hypercube sampling is performed for five different lower and upper bound priors of the parameters and training is performed. A) Percentage of successful convergence of an example model, where success is defined as the percentage of models below the loss threshold on the x-axis. B) A similar plot, but now with a fixed prior and five SBML models. C) Heatmap for the initialization success percentage (upper diagonal) and training success (lower diagonal) for $loss<10^{-3}$. The number of successfully trained models is dependent on the initialization success and is therefore always a lower percentage. D,E,F) Examples of fitted SBML models after training.

To motivate the main analysis of SBML models, we show the influence of parameter bounds on one model when sampling 100 initializations using Latin Hypercube Sampling (Fig 2A) [72]. The percentage of models that are below the loss threshold are reported for five different parameter bound priors. It is observed for most models that with larger bounds, the percentage of succesfully trained models is either negatively affected or not affected. This behavior is expected, as starting your parameter initialization closer to the true optimum would be an easier problem. We also compare the convergence success among five different systems biology models with a fixed prior bound ($\frac{1}{10}\theta_{true}\leq \theta_{true}\leq 10\theta_{true}$) (Fig 2B). This allows for comparing the performance of the parametrization framework across a large collection of Systems Biology models. In order to compare many models across different bounds, we chose a loss threshold of 10^−3^.

### Initialization success and the training process is stable across models and priors

Fig 2C shows a heatmap for 25 SBML models for five different priors. The left column of each model is the initialization success percentage, while the right column is the percentage of models after training that were below the loss threshold (10^−3^). Overall, we see a high initialization success percentage for most SBML models. For many models, the loss function can be calculated independent of the bounds used in this study. For six models, the behavior of decreasing initialization success given the priors is observed, in line with what would be expected [47,54,56,58,68,70]. Interestingly, a reversed pattern is observed for two models, where the initialization success increases with larger bounds [66,69]. For one model, the initialization success is low, independent of the prior [50].

The training process consists of 3000 iterations of stochastic gradient descent using AdaBelief and a global norm clipping with a learning rate of 10^−3^, without any batching of training data [38,40]. The right column of each model for Fig 2C shows the percentage of successfully trained parameter given the initialization success. Here, the effect of priors is observed more clearly. For the smallest bounds ($\left[1/2\theta_{true},2\theta_{true}\right]$), we observe good convergence to the global minimum for most models. In some cases [70], the loss during the initialization is already below the chosen loss threshold, therefore having a similar percentage of successful initializations to successful training convergence. Parameter initializations that are not successfully trained can occur due to several reasons. First, the number of steps of the optimizer might not be enough for the loss to be below the threshold. Second, during optimization the gradient descent might lead to a parameter set that is not numerically solvable due to stiffness or divergence issues. When we increase the parameter bounds, it is typically observed that a decreasing number of parameter initializations are successfully trained. Generally speaking, we do not observe a clear relation between the number of parameters (or state variables) and the difficulty of training these models, except that the computation time increases for larger scale models.

To further observe whether the models fit data such that it properly captures the metabolite concentration dynamics, we simulate three models with losses below the threshold given in the heatmap for their best fit parameters with bounds $\left[\frac{1}{10}\theta_{true},10\theta_{true}\right]$ and compare it to the simulated data points (Fig 2D–F). It can be observed that for the model from Beer *et al*. (2014) and Messiha *et al*. (2014) the dynamics are fairly close if not perfectly matching the simulated data points (Fig 2D, E). For the model from Garde *et al*. (2020), despite the loss being below the threshold, the trained dynamics are not close to the true dynamics, but rather finds a heavily oscillating parameter set that then matches the data. This behavior is not observed when the prior is between one-fifth and five of the true parameters. This suggests that evaluating the fit only based on the objective can be misleading and visual inspection of the dynamics might be preferred. Furthermore, for some models prior information could be more important than for others, and methods to filter parameters based on spectral analysis of the Jacobian matrix [18,20] could be beneficial.

Overall, initialization success is stable across a wide variety of models, which increases the likelihood of effectively learning parameters. While we see for some models a dependency on the bounds, this effect can be mitigated by increasing the initial sampling size. The loss after training shows a clearer effect of the priors. This indicates that when parameterizing kinetic models, the prior can be of high importance. Although it might in practice be difficult to define strict bounds for parameters *a priori*, using kinetic databases like Brenda might be a way to guess an initial parameter. Additionally, analysis of sloppiness in systems biology, as well as identifiability analysis, might aid in increasing success of model fitting (see Fig D in S1 text) [12,74].

### Large scale kinetic models can be trained using *jaxkineticmodel*: an example of glycolysis

While we have shown that neural differential equations could be used for a large variety of different SBML models, real time-series metabolomics data has additional complexity in terms of heterogeneity of the measured metabolites as well as noise. We therefore reimplemented a kinetic model of glycolysis in *JAX* [33,73]. This model consists of 20 metabolites, 37 reactions, and 141 parameters. The kinetic mechanisms used are implemented as *JAX* compatible classes for well-known equations (e.g., Michaelis Menten) that can be reused for other purposes (see Table A in S1 text).

**Fig 3:**
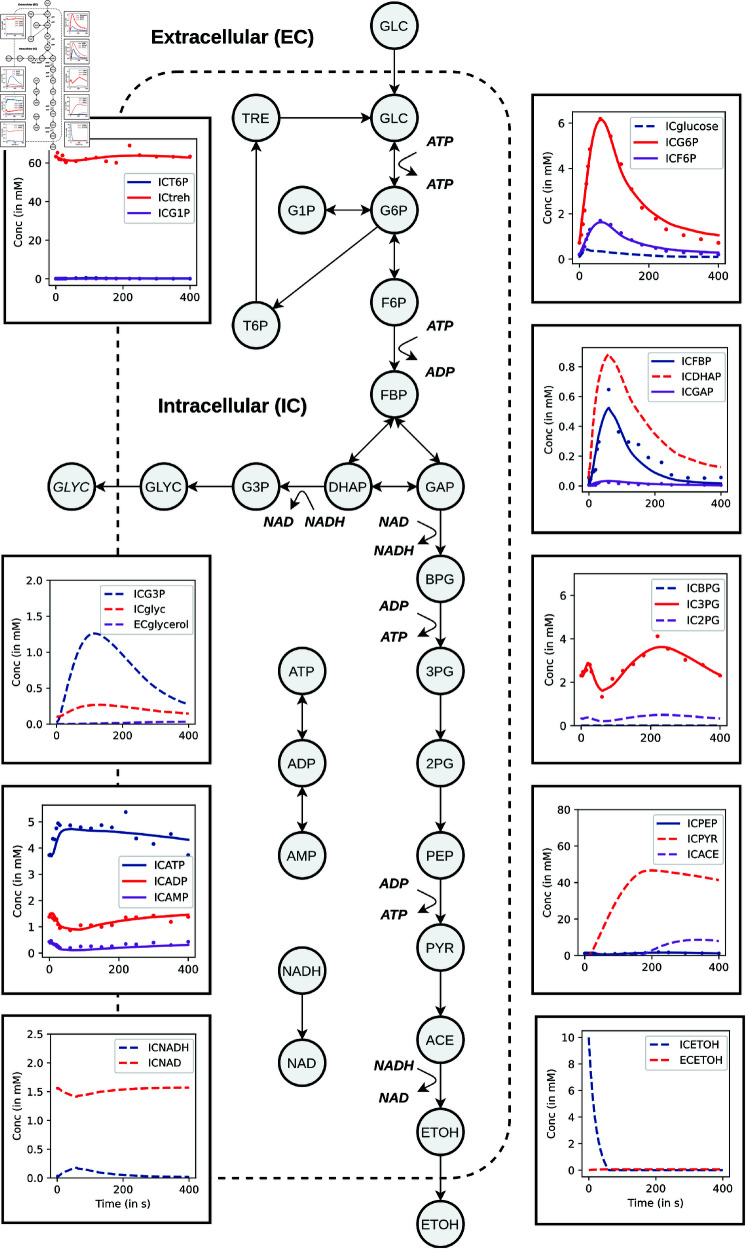
Fitting a feast/famine cycle to a glycolysis model. A simplified schematic of glycolysis, along with panels of the dynamic response of glycolysis to a glucose pulse [39]. Intracellular (IC) and extracellular (EC) metabolite concentrations that were measured are shown as dots in the panels, while lines indicate model predictions. Metabolites that were included in the model, but for which there was no available data, are represented as dashed lines.

The model was initialized with literature values reported in the previous MATLAB implementation [73]. 10000 iterations of stochastic gradient descent using AdaBelief were performed, resulting in the fit as reported in Fig 3. The panels show the dynamic response of a glucose pulse during a feast-famine cycle for some previously reported datasets [39]. The striped lines are inferred dynamic responses of metabolites, for which no training data was available. The glucose pulse obviously leads to an increase through the upper- and lower glycolysis, as well as a pulse through the glycerophospholipid pathway. In the first steps ATP is formed, which is well-captured by the dynamic response of the model. There is also a drop in NAD+ due to an increased activity through the lower glycolysis, where NAD+ is consumed by *glyceraldehyde-3-phosphate dehydrogenase*.

Overall, a good fit is observed between modeled and measured data. Even though this is a relatively large and complicated model of glycolysis, the fitting process for one dataset took approximately four hours (for 10000 update steps) on one CPU with 10GB memory. This shows that the implementation could be used to fit large-scale kinetic models. Furthermore, the training process is easy to extend to fit multiple datasets simultaneously (see Fig C in S1 text).

### Flexibility of automatic differentiation with *jaxkineticmodel* allows for hybridizing kinetic models with neural ODEs.

Although *jaxkineticmodel* is capable of parameterizing large kinetic models, several other parameterization methods reported in the literature can perform similarly for mechanistic models (see S1 text). One package that is methodologically similar to *jaxkineticmodel* is PyPESTO with gradient computations from AMICI [23,24]. Instead of automatic differentiation, AMICI uses symbolically derived parameter sensitivity equations in combination with the efficient CVODE solver to perform the forward and adjoint simulation [53]. This results in PyPESTO/AMICI outperforming *jaxkineticmodel* in terms of computation time, which can primarily be attributed to the use of CVODE (see Tables B and C in S1 text). However, integrating neural networks with mechanistic models, an aspect that is of increasing interest to the scientific machine learning community when modeling biological systems [30–32], becomes practically impossible when the neural network components need to be symbolically derived.

**Fig 4:**
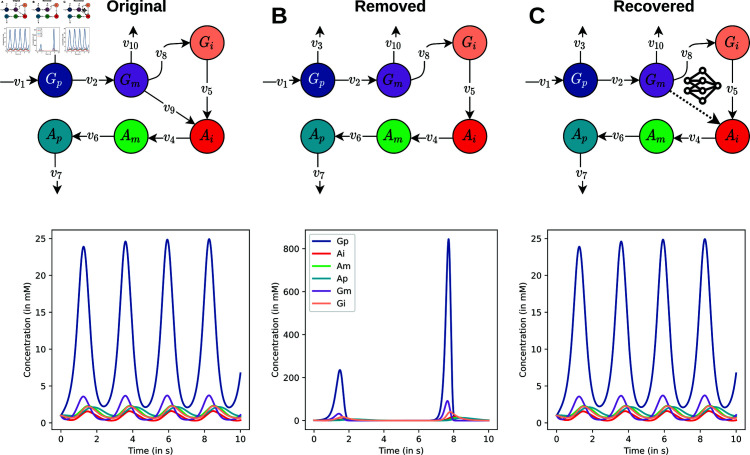
Jaxkineticmodel allows for hybridizing kinetic models with neural networks. Due to automatic differentiation capabilities of JAX/Diffrax, integrating neural networks with kinetic models becomes straightforward. As an example, we show how kinetic mechanisms can be replaced with a neural network. A) A schematic representation (upper) and time-series plot (lower) of a minimal model of metabolic oscillations in a biofilm [44]. B) Schematic and time-series plot of the same model without $v_{9}$. C) The hybrid kinetic model, where $v_{9}$ is replaced with a neural network. The time-series plot shows the dynamics of the hybrid model after training.

To present this flexibility of automatic differentiation, we show an example of how mechanistic models can be combined with neural networks, using a minimal model of metabolic oscillations in biofilms [44]. The original model consists of six species and ten reactions with an oscillatory dynamic (Fig 4A). Suppose that a stoichiometry and mechanism of a reaction are unknown, for example the reaction that transforms *G*_*m*_ into *A*_*i*_ ($v_{9}$). This missing reaction mechanism changes the dynamic behavior of the model (Fig 4B). By hybridizing the kinetic model with a neural network for $v_{9}$, the dynamic behavior of the original model can be recovered after training (Fig 4C). This can be performed for all reactions in the model, where for eight out of ten reactions the expected dynamic behavior could be recovered (Fig F in S1 text). In the other two reactions, the dynamics of the masked model is diverging and get stuck in a local minimum when training. Further work is required to understand how training hybrid models can be successful in all cases.

## Availability and future directions

Large-scale kinetic models have the potential to explain systems level behavior of biological systems, but their parametrization has proven to be challenging [10]. Furthermore, the dynamics of biological systems are often only partially understood from a mechanistic perspective, stressing the need for hybrid approaches [30–32]. In this work, we have implemented a training framework, inspired by techniques from neural ODEs, tailored to systems biology models. Due to the *JAX*-based simulation using *Diffrax*, the training is relatively fast, which paves the way to large-scale kinetic model training [33,34]. Furthermore, due to support for Neural ODEs by *Diffrax*, flexibility in using mechanistic and neural network approaches offers a useful approach to modeling biological systems.

Our default implementation of the *jaxkineticmodel* training framework mitigates parameter fitting challenges caused by characteristics of biological systems [11–13], as well as solutions from the field of neural ODEs [15,27,38]. As fitting kinetic models often requires a multiple starting approach, we note that the current approach is easy to parallelize, and will be a future direction. The implementation is publicly available as a Python package at https://github.com/AbeelLab/jaxkineticmodel. All data, source code, and results are available on the 4TU repository (https://data.4tu.nl/datasets/3662eca5-7077-4ca3-8f66-d051e2c79cbe).

We showcase the methodology on a large collection of SBML models of varying sizes [11,43] and perform a principled analysis on the effect of parameter priors on the performance. We furthermore show that *jaxkineticmodel* is useful for parameterizing large kinetic models in real-world applications by fitting a glucose pulse dataset from a feast/famine experiment to a previously established glycolysis model [39,73]. Finally, we show that due to automatic differentation capabilities in *JAX*, kinetic models can be hybridized with neural network components when a systems is only partially understood [30,31]. This feature makes *jaxkineticmodel* different from other parameterization methods. Future work will aim to make these hybrid modeling capabilities more accessible to the user through tutorials and documentation.

## Supporting information

S1 Text Supporting information on neural ordinary differential equations and *jaxkineticmodel.* This contains Figs **A–F** and Tables **A–D**
(PDF)
