# Supplementary material for: Jaxkineticmodel: Neural ordinary differential equations inspired parameterization of kinetic models
Source: PLoS Comput Biol. 2025 Jul 7;21(7):e1012733. doi: 10.1371/journal.pcbi.1012733 (PMC12258584; doi:10.1371/journal.pcbi.1012733)
Supplement: S1 Text — This contains Figs A–F and Tables A–D (PDF) [file pcbi.1012733.s001.pdf]

Supporting Information for *Jaxkineticmodel*: Neural Ordinary Differential Equations Inspired Parameterization of Kinetic Models

Paul van Lent[a], Olga Bunkova[a], Bálint Magyar[a], Léon Planken[a], Joep Schmitz[b], Thomas Abeel[a,c]

[a] *Delft University of Technology, van Mourik Broekmanweg, Delft, 2628 CN Delft, Zuid Holland, Netherlands*
[b] *dsm-firmenich, Alexander Fleminglaan 1, Delft, 2613AX, Zuid Holland, Netherlands*
[c] *Broad Institute of MIT and Harvard, Cambridge, MA02142, Massachusetts, United States of America*

1. Neural Ordinary Differential Equations

Neural ordinary differential equations are differential equations where the right-hand side is defined by a neural network (eq. 1) [1].

$$\frac{dm(t)}{dt} = NN(t, m(t), \theta) \tag{1}$$

Here, $m(t)$ is the metabolic state at time t and the parameters (weights and biases) θ that parameterize the neural network. One could solve $\frac{dM}{dt}$ on the time interval $[0, T]$ (eq. 2) as an initial value problem using a numerical solver (eq. 3). This could for example be done for multiple evaluated timepoints in a time-series datasets, such as for changing metabolite concentrations.

$$m(T) = m(0) + \int_0^T NN(t, m(t), \theta)dt \tag{2}$$

$$m(T) \approx odesolver(m(0), T, NN, \theta) \tag{3}$$

Suppose we want to model a dataset of observed metabolic states over time with N observed data points. We use the metabolic states $m(t)$ at $t = 0$ as the initial values, an initial guess of θ and a predefined neural network architecture to solve the initial value problem. The predicted dataset can be compared to the observed data using a loss function (4).

$$J(m_{pred}, m_{obs}) = \frac{1}{N}\sum_N (m_{pred} - m_{obs})^2 \tag{4}$$

Using any gradient descent algorithm, one can improve the fit of the observed and the predicted data, which requires calculation of the gradients of the loss function with respect to the parameters ($\frac{dL}{d\theta}$). Multiple methods exist to calculate these gradients, but the most efficient ones are through calculating adjoint sensitivities [1, 2].
For the purpose of our problem (kinetic models), there is no theoretical difference between gradient calculation when the right-hand-side is a neural network compared to the right-hand side often used in kinetic modeling.

2. Gradients in neural ODEs

Gradients can be calculated in roughly three ways: 1) forward sensitivity analysis, 2) adjoint sensitivity through optimize-then-discretize, and 3) adjoint sensitivity through discretize-then-optimize (Fig. A). Forward sensitivity analysis requires solving the system of ODEs $|p+1|$ times, where small perturbations to the parameters are used to calculate the effect of the parameter on the loss. For small systems, forward sensitivity analysis works well. For larger systems with many parameters, the adjoint sensitivity methods are more efficient, as they require solving the system of ODEs only two times: one forward pass and one backward pass.

Below, we will report on the discretize-then-optimize scheme of gradient estimation, since this one is used in the paper. For a treatment of forward sensitivity analysis and the optimize-then-discretize method, we refer to the following paper [3].

The loss function is depending on the initial state m and the parameters θ. The gradients $\frac{dJ}{d\theta}$ are the integral of the loss function (eq. 5).

$$\frac{dJ}{d\theta} = \int_0^T \frac{d}{d\theta} J(m, \theta) \tag{5}$$

$$= \int_0^T \frac{\partial J}{\partial \theta} + \frac{\partial J}{\partial m}\frac{dm}{d\theta} dt \tag{6}$$

The first term captures the explicit dependence of the loss function on θ, while the second term captures the implicit dependence of the loss function on θ through the state $m(t)$ (which is in itself dependent on θ through the ODE solve). Here, $\frac{\partial J}{\partial m}$ is the sensitivity of the loss with respect to the state $m(t)$ and $\frac{dm}{d\theta} \in \mathbf{R}^{|\mathbf{m}|\times|\theta|}$ describes the sensitivity of the state $m(t)$ with respect to the parameters θ. This latter one is termed the Jacobian and we need this in order to get gradients.

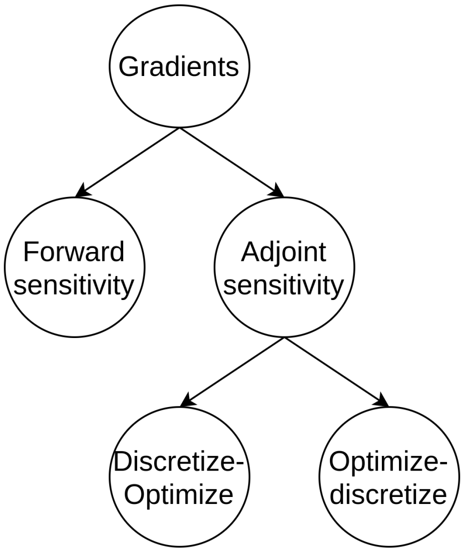

Fig. A: Scheme of the methods to estimate the gradients $\frac{dL}{d\theta}$

2.1. Adjoint sensitivity analysis: the discretize-then-optimize scheme

2.1.1. The discretize-then-optimize scheme

Suppose we have a system of ODEs like before, which could be a neural network or kinetic model, here termed f (eq. 7).

$$\frac{dm(t)}{dt} = f(m(t), t, \theta) \tag{7}$$

We can solve this using a numerical solver, where the simplest method would be the Euler method (eq. 8).

$$m_{n+1} = m_n + \Delta t \cdot f(m_n, t_n, \theta) \tag{8}$$

The forward pass is thus defined as a number of discrete steps on the interval $T = [t_0, t_1, ..., t_N]$ (eq. 10).

$$m_1 = m_0 + \Delta t \cdot f(m_0, t_0, \theta) \tag{9}$$
$$m_2 = m_1 + \Delta t \cdot f(m_1, t_1, \theta) \tag{10}$$
$$m_3 = m_2 + \Delta t \cdot f(m_2, t_2, \theta) \tag{11}$$
$$\vdots \tag{12}$$
$$m_N = m_{N-1} + \Delta t \cdot f(m_{N-1}, t_{N-1}, \theta) \tag{13}$$

For the backward pass, automatic differentiation can be applied to compute the gradient of the loss J with respect to the parameters θ and the initial values m_0, which is done by backpropagating through the steps. This is sometimes referred to as backpropagation through time (BPTT) [4]. One can thus view an ODE as a residual neural network, where the number of layers N is determined using an ODE solver. One can apply the chain rule in a recursive manner, similar to how backpropagation is performed through neural network layers (eq. 14).

$$\frac{dJ}{d\theta} = \sum_{n=0}^{N-1} \frac{\partial J}{\partial m_N} \frac{\partial m_N}{\partial m_n} \frac{\partial m_n}{\partial \theta} \tag{14}$$

To get the gradient with respect to the loss, one thus has to 1) backpropagate through time-steps and 2) backpropagate through parameters θ.

Backpropagate through time steps. We start by computing gradients recursively using backpropagation through the sequence of discretized timesteps (from m_n to m_0). The update rule (when we use the Euler method) is

$$m_{n+1} = m_n + \Delta t \cdot f(m_n, t_n, \theta) \tag{15}$$

We can take derivative w.r.t. m_n and get

$$\frac{\partial m_{n+1}}{\partial m_n} = 1 + \Delta t \cdot \frac{\partial f(m_n, t_n, \theta)}{\partial m_n} \tag{16}$$

Now, one applies the chain rule for backpropagation, which leads to the gradients of the loss with respect to m_n

$$\frac{\partial J}{\partial m_n} = \frac{\partial J}{\partial m_{n+1}} \frac{\partial m_{n+1}}{\partial m_n} \tag{17}$$

Backpropagate through parameters The gradients w.r.t. θ can also be computed using the chain rule

$$\frac{\partial m_{n+1}}{\partial \theta} = \Delta t \cdot \frac{\partial f(m_n, t_n, \theta)}{\partial \theta} \tag{18}$$

Then, the total gradient with respect to θ is given by:

$$\frac{dJ}{d\theta} = \sum_{n=0}^{N-1} \frac{\partial J}{\partial m_{n+1}} \cdot \Delta t \cdot \frac{\partial f(m_n, t_n, \theta)}{\partial \theta} \tag{19}$$

Note that this only requires two solves: one for the forward system (to get the loss function) and a backwards solve, where evaluated time points are used to establish a gradient. This is a crucial aspect of the adjoint sensitivities; it does not grow with the number of parameters, making it more scalable to large systems of ODEs. This version of computing adjoint sensitivities was used in this paper and is the default in *Diffrax*[2]. While not quantified, we have noticed that this computation through the internals of the solver is a more stable way to compute gradients then the optimize-then-discretize version that was popularized in the original neural ODE paper [1].

3. *JAX*-based Kinetic Models

3.1. Introduction

Metabolic kinetic models are systems of Ordinary Differential Equations (ODEs) that are typically of the form (eq. 20)

$$\frac{dm(t)}{dt} = S \cdot v(t, y, \theta) \tag{20}$$

Here, $S \in \mathbb{R}^{m \times n}$ represents the stoichiometric matrix that describes the mass balances for the m metabolites given the n reaction fluxes and v represents a vector of flux functions that is dependent on t, the concentration of metabolites y_0, and a set of biologically inspired parameters θ. An example of a flux function is the Michaelis-Menten equation, which represents the irreversible conversion of $S \to P$ (eq. 21), but many (complicated) mechanisms exist to represent a variety of chemical reactions.

$$v_i = V_{max} \cdot \frac{[S]}{K_S + [S]} \tag{21}$$

$$\vec{\theta} = [V_{max}, K_S] \tag{22}$$

Many mechanisms have functional forms that could be shared between different reactions, albeit with different parameter values. Therefore, we have implemented a large set of kinetic mechanisms to be compatible with *JAX* [5].

3.2. Table of mechanisms

We implemented more than twenty *JAX*-compatible kinetic mechanisms that were required for modeling glycolysis [6] (Table. A). These cover many qualitative stoichiometric mechanisms and (ir-)reversibility characteristics and can be used to also model other metabolic pathways. For some enzymes, very specific mechanisms are

required due to inhibitory interactions and other tight regulations that are essential to proper functioning of glycolysis.

Table A: **Overview of the reactions used in this study.** The types of reactions, their names in *JAX* class, the number of parameters, and the reactions where they are used are listed. Some enzyme-specific (such as *Alcohol Dehydrogenase* and *AMP deaminase* are implemented but not reported in the table.

Type of Reaction	Name (in JAX class)	Number of Parameters	Used in
$A \to B$	Jax_MM_Irrev_Uni	2	vNTH1, vmitoNADH, vATPmito
$A \to B$	Jax_MM_Irrev_Uni_w_modifiers	2 + modifiers	vHOR2
$A \leftrightarrow B$	Jax_MM_Rev_UniUni	4	vTPI1, vENO, vPGM, vPGM1, vPGI
$A \to$	Jax_MM_Sink	2	vsink*
$A \to$	Jax_MA_Irrev	1	vISN1, vPNP1, vHP1, vADE1312
$A \Rightarrow$	Jax_Facilitated_Diffusion	3	vGLT
$A \Rightarrow$	Jax_Diffusion	2	vETOHtransport, vGLYCEROLtransport
$A + B \to C$	Jax_MM_Irrev_Bi	3	vATPmito
	Jax_MM_Irrev_Bi_w_Inhibition	3	vTPS2
	Jax_MM_Irrev_Bi_w_modifiers	3 + modifiers	vTPS1
$A + B \leftrightarrow C + D$	Jax_MM_Rev_BiBi	6	vPGK
	Jax_MM_Rev_BiBi_w_Inhibition	7	vHXK
	Jax_MM_Rev_BiBi_w_Activation	9	vG3PDH
$A + B \leftrightarrow C + D$	Jax_MA_Rev_Bi	2	vADK
$A \leftrightarrow B + C$	Jax_MM_Rev_UniBi	5	vALD
$A + B \to C + D + E$	Jax_MM_Ordered_BiTri	7	vGAPDH, vPFK
$A + B \to C + D$	Jax_ADH	15	vPFK, vPYK
$A \to B + C$	Jax_Hill_Bi_Irreversible_Activation	7	vPYK, vPDC
$A \to B + C$	Jax_Hill_Irreversible_Inhibition	7	vPDC, vADH

4. Comparing *jaxkineticmodel* with other popular parameterization methods

Several parameter estimation tools already exist for kinetic models. Roughly speaking, they can be categorized based on the following characteristics: 1) the method is specifically developed for steady state data (e.g., fluxomics, metabolomics, proteomics) or dynamic data. 2) The method uses gradients to optimize or is gradient-free. Below, we have reported a categorization of several parameterization methods with a short description.

- K-FIT [7]: a gradient-based, steady state method. Minimizes the objective function between observed fluxes and predicted fluxes.
- KETCHUP [8]: a gradient-based, steady state method. This method builds on top of K-FIT, but uses a semi-automated way of formulating the kinetic model and uses an interior point optimizer for parameterization.

- ORACLE [9]: a gradient-free, steady state method. Uses a Monte Carlo sampling approach for back-calculating parameters from a constraint-based model.

- REKINDLE [10]: a gradient-free, steady state method. REKINDLE uses ORACLE to produce parameter sets, then categorizes the parameter sets based on physiological relevance. It then uses a Generative Adversarial Network (GAN) to learn the distribution of the physiologically relevant parameter space. This method is mostly useful for retraining on small datasets, when the GAN is already available.

- RENAISSANCE [11]: a gradient-free, steady state method. Uses an evolutionary algorithm to choose new parameter sets that are physiologically relevant.

- MAUD [12]: a gradient-free method for steady-state and (potentially) dynamic data. MAUD uses a Bayesian approach, while incorporating thermodynamic constraints, to parameterize parameters. A key advantage of MAUD is that parameter uncertainties are given by the posterior distribution.

- AMICI/PyPESTO [13, 14]: a gradient-based method for steady-state and dynamic data. AMICI compiles the sensitivity equations for the kinetic model to *C* and PyPESTO offers an interface to AMICI and many popular optimizers that use the sensitivity equation to do gradient descent.

Of these methods, AMICI/PyPESTO is the closest methodologically to *jaxkineticmodel*. Similarly to *jaxkineticmodel*, it offers optimizers based on gradient descent with adjoint and forward sensitivity analysis methods for computing gradients. AMICI/pyPESTO differs in that for a given model it first determines the sensitivity equations symbolically and compiles the model to *C++*. These sensitivity equations can be used to calculate gradients of the loss with respect to parameters [13]. It also provides an interface to the CVODE solver: a specialized stiff solver particularly effective for biochemical systems [16]. This symbolic derivation and solver makes PyPESTO/AMICI computationally fast. However, due to this symbolic derivation of the sensitivity equations, there is less flexibility in terms of hybridizing kinetic models with neural ODEs, as is done in recent work in universal differential equations [29, 27, 28]. *Jaxkineticmodel* offers an advantage here in that it uses automatic differentiation, which is more flexible in what it can use for optimization, as we reported in the main results and figure F. This flexibility may however come at the expense of computation time. To quantify this, we have compared relevant aspects of the forward simulation and gradient computation between PyPESTO/AMICI and *jaxkineticmodel*.

4.1. Time comparison between jaxkineticmodel and PyPESTO/AMICI

As PyPESTO/AMICI is a highly configurable and versatile tool for fitting kinetic models, it is not straightforward to perform time comparisons for a full optimization process. There are many parameter initialization methods, optimizers, objective functions, and other hyperparameters of the optimization process that lead to flawed comparisons. To ensure a good comparison between *jaxkineticmodel* and PyPESTO, we first introduce where the main computational bottlenecks are in *jaxkineticmodel* and then compare these aspects.

The stochastic gradient descent uses the gradients of the loss to update the parameters (Algorithm 1). There are two computationally expensive evaluations: the calculation of the loss (Algorithm 1, line 1) and the calculation of the gradients of the loss (Algorithm 1, line 2). The other aspects of the update rule have a negligible impact on computation time. Furthermore, comparing across different optimizers may lead to unfair comparisons as the number of update steps may vary between methods. We therefore focus on the loss and gradient computation. To compare these between PyPESTO and *jaxkineticmodel*, we chose five models with differing number of parameters.

Algorithm 1 Update Parameters

Require: Optimizer state `opt_state`, parameters `params`, inputs `ts`, targets `ys`, loss function `loss_func`, optimizer `optimizer`

Ensure: Updated optimizer state, parameters, loss, gradients

1: `loss` ← `loss_func(params, ts, ys)` {This may be computationally expensive}
2: `grads` ← `grad(loss_func)(params, ts, ys)` {This may be computationally expensive}
3: `(updates, opt_state)` ← `optimizer.update(grads, opt_state)`
4: `params` ← `apply_updates(params, updates)`
5: **return** `opt_state, params, loss, grads`

4.1.1. Time comparison forward simulation

For the loss function, the dominating computational step is solving the ODEs, which is required to calculate the loss function. Below, we report a time comparison between two solvers from *Diffrax* and the CVODE solver used in AMICI [13, 16]. We use similar error tolerances and other solver hyper-parameters. As can be seen, CVODE outperforms in terms of computation time in every instance. This can be attributed to the fact that CVODE is a high-performance solver, working specifically well for stiff ODEs.

Table B: **Time comparison of solvers across models (in s) (mean ± std).** Standard deviations are the result of simulating each model for 100 parameter sets, repeated 10 times each (1000 solves). Kvaerno5 and Dopri5 are solvers implemented in Diffrax, and CVODE is implemented in AMICI/PyPESTO.

**Model**	**Parameters**	**Kvaerno5 (Diffrax)**	**Dopri5 (Diffrax)**	**CVODE**
Smallbone *et. al (2013)*	10	0.0098 ± 0.0009	0.0035 ± 0.0003	**0.0024 ± 0.0002**
Becker *et. al (2010)*	17	0.0055 ± 0.0005	0.0033 ± 0.0003	**0.0021 ± 0.0002**
Fujita *et. al (2010)*	26	0.0056 ± 0.0003	1.2757 ± 0.0196	**0.0020 ± 0.0002**
Raia *et. al (2011)*	45	0.0068 ± 0.0003	0.0036 ± 0.0003	**0.0022 ± 0.0002**
Messiha *et. al (2013)*	192	0.1127 ± 0.0070	0.3522 ± 0.0093	**0.0197 ± 0.0006**

4.1.2. Gradient computation (adjoint method)

The gradient calculation is the more expensive calculation when updating parameters during gradient descent. We perform a similar experiment as above for two different solvers from Diffrax (Kvaerno5 and Dopri5) and the forward and adjoint method implemented in AMICI. Overall, the AMICI adjoint state method outperforms in every scenario in terms of computation time. The adjoint state method is more efficient compared to AMICI's forward sensitivity method, as mentioned in section SI2 and also reported in [26]. For future work, implementation of CVODE in diffrax would be highly valuable, as this might lead to faster computation of gradients, while also keeping the flexibility that automatic differentiation offers.

Table C: **Time comparison of gradient computation**. We tested the forward and adjoint method of AMICI and compared it to the adjoint method used in Diffrax/*jaxkineticmodel* for two different solvers. Each model was run for 100 parameter sets, repeated 10 times (1000 solves).

**Model**	**Kvaerno5 (Diffrax)**	**Dopri5 (Diffrax)**	**AMICI forward**	**AMICI adjoint**
Smallbone *et. al (2013)*	0.0492 ± 0.0024	0.0240 ± 0.0015	0.0149 ± 0.0059	**0.0109 ± 0.0041**
Becker *et. al (2010)*	0.0316 ± 0.0016	0.0183 ± 0.0026	0.0076 ± 0.0035	**0.0014 ± 0.0003**
Fujita *et. al (2010)*	0.0760 ± 0.0054	NaN	0.0338 ± 0.0126	**0.0028 ± 0.0006**
Raia *et. al (2011)*	0.0417 ± 0.0017	0.0368 ± 0.0049	0.0222 ± 0.0021	**0.0034 ± 0.0004**
Messiha *et. al (2013)*	0.9914 ± 0.0164	14.4700 ± 1.5348	1.3980 ± 0.3910	**0.4189 ± 0.0526**

5. Systems Biology Markup Language support

SBML compatibility of *jaxkineticmodel* is a feature that we aim to expand to as many model cases as possible. To measure the SBML compatibility of *jaxkineticmodel*, we used the SBML test-suite semantic test cases to quantify the support (500 models) [18]. We compared the number of models for their simulations against libroadrunner [19]. Overall, *jaxkineticmodel* matches libroadrunner in terms of the fraction of

similar simulations (see Table D). Some specific mathematical notations are not yet supported, but will be provided in the future. A previous implementation of a jax-based simulation framework, SBMLtoODEjax, supports 22% of the SBML models [30].

SBML level	Similar Simulation	Failed Simulation	Discrepancies
jaxkineticmodel (l2v1)	0.59	0.24	0.18
libroadrunner (l2v1)	0.64	0.21	0.15
jaxkineticmodel (l2v2)	0.55	0.29	0.16
libroadrunner (l2v2)	0.64	0.20	0.16
jaxkineticmodel (l2v3)	0.55	0.29	0.16
libroadrunner (l2v3)	0.64	0.20	0.16
jaxkineticmodel (l2v4)	0.56	0.77	0.42
libroadrunner (l2v4)	0.61	0.55	0.44
jaxkineticmodel (l3v1)	0.39	0.47	0.14
libroadrunner (l3v1)	0.45	0.32	0.23
jaxkineticmodel (l3v2)	0.37	0.50	0.13
libroadrunner (l3v2)	0.43	0.35	0.22

Table D: SBML compatibility support

6. Glycolysis model

The yeast glycolysis model was retrieved from a previously established and validated model [6]. All kinetic mechanisms were re-implemented to be compatible with *JAX/Diffrax* [2, 5].

6.1. Mass balances

The system of equations is shown below and was retrieved from [1]. Some differences in the modeling and fitting process are reported below the mass balances. In total, 29 metabolite ODEs were set up with 38 reactions and 141 parameters describing the time evolution of the glucose pulse through glycolysis. The model can be found in the Github repository (models/manual_implementations).

$$\frac{d[\mathrm{GLCi}]}{dt} = -v_{\mathrm{HXK}} + v_{\mathrm{GLT}} + 2 \cdot v_{\mathrm{NTH1}};$$

$$\frac{d[\mathrm{G6P}]}{dt} = +v_{\mathrm{GLK}} - v_{\mathrm{PGI}} + v_{\mathrm{sinkG6P}} + v_{\mathrm{PGM1}} - v_{\mathrm{TPS1}};$$

$$\frac{d[\mathrm{G1P}]}{dt} = -v_{\mathrm{PGM1}} - v_{\mathrm{UGP}};$$

$$\frac{d[\mathrm{T6P}]}{dt} = +v_{\mathrm{TPS1}} - v_{\mathrm{TPS2}};$$

$$\frac{d[\mathrm{TRE}]}{dt} = +v_{\mathrm{TPS2}} - v_{\mathrm{NTH1}};$$

$$\frac{d[\mathrm{F6P}]}{dt} = -v_{\mathrm{PFK}} + v_{\mathrm{PGI}} + v_{\mathrm{sinkF6P}};$$

$$\frac{d[\mathrm{F16BP}]}{dt} = +v_{\mathrm{PFK}} - v_{\mathrm{ALD}};$$

$$\frac{d[\mathrm{GAP}]}{dt} = +v_{\mathrm{ALD}} - v_{\mathrm{GAPDH}} + v_{\mathrm{TPI1}} + v_{\mathrm{sinkGAP}};$$

$$\frac{d[\mathrm{DHAP}]}{dt} = +v_{\mathrm{ALD}} - v_{\mathrm{TPI1}} - v_{\mathrm{G3PDH}};$$

$$\frac{d[\mathrm{G3P}]}{dt} = +v_{\mathrm{G3PDH}} - v_{\mathrm{HOR2}};$$

$$\frac{d[\mathrm{GLYCEROL}]}{dt} = +v_{\mathrm{HOR2}} - v_{\mathrm{GLYCEROLtransport}};$$

$$\frac{d[\mathrm{BPG}]}{dt} = +v_{\mathrm{GAPDH}} - v_{\mathrm{PGK}};$$

$$\frac{d[\mathrm{P3G}]}{dt} = +v_{\mathrm{PGK}} - v_{\mathrm{PGM}} + v_{\mathrm{sinkP3G}};$$

$$\frac{d[\mathrm{P2G}]}{dt} = +v_{\mathrm{PGM}} - v_{\mathrm{ENO}};$$

$$\frac{d[\mathrm{PEP}]}{dt} = +v_{\mathrm{ENO}} - v_{\mathrm{PYK}} + v_{\mathrm{sinkPEP}};$$

$$\frac{d[\mathrm{PYR}]}{dt} = +v_{\mathrm{PYK}} - v_{\mathrm{PDC}} + v_{\mathrm{sinkPYR}};$$

$$\frac{d[\mathrm{ACE}]}{dt} = +v_{\mathrm{PDC}} - v_{\mathrm{ADH}} + v_{\mathrm{sinkACE}};$$

$$\frac{d[\mathrm{ETOH}]}{dt} = +v_{\mathrm{ADH}} - v_{\mathrm{ETOHtransport}};$$

$$\frac{d[\mathrm{ATP}]}{dt} = +v_{\mathrm{ADK1}} - v_{\mathrm{GLK}} - v_{\mathrm{ATPase}} - v_{\mathrm{PFK}} + v_{\mathrm{PGK}} + v_{\mathrm{PYK}} - v_{\mathrm{TPS1}} + v_{\mathrm{mito}};$$

$$\frac{d[\mathrm{ADP}]}{dt} = -2 \cdot v_{\mathrm{ADK1}} + v_{\mathrm{GLK}} + v_{\mathrm{ATPase}} + v_{\mathrm{PFK}} - v_{\mathrm{PGK}} - v_{\mathrm{PYK}} + v_{\mathrm{TPS1}} - v_{\mathrm{mito}};$$

$$\frac{d[\mathrm{AMP}]}{dt} = +v_{\mathrm{ADK1}} - v_{\mathrm{Amd1}} + v_{\mathrm{Ade13}} - v_{\mathrm{Ade12}};$$

$$\frac{d[\mathrm{PI}]}{dt} = -v_{\mathrm{GAPDH}} + v_{\mathrm{ATPase}} + v_{\mathrm{HOR2}} + v_{\mathrm{RHR2}} + 2 \cdot v_{\mathrm{TPS1}} + v_{\mathrm{TPS2}} - v_{\mathrm{mito}}$$
$$+ v_{\mathrm{Isn1}} - v_{\mathrm{Pnp1}} + v_{\mathrm{vacuolePi}} - v_{\mathrm{sinkG6P}} - v_{\mathrm{sinkF6P}} - v_{\mathrm{sinkGAP}} - v_{\mathrm{sinkP3G}} - v_{\mathrm{sinkPEP}};$$

$$\frac{d[\mathrm{IMP}]}{dt} = +v_{\mathrm{Amd1}} - v_{\mathrm{Ade13}} - v_{\mathrm{Ade12}} + v_{\mathrm{Hpt1}} - v_{\mathrm{Isn1}};$$

$$\frac{d[\mathrm{INO}]}{dt} = +v_{\mathrm{Isn1}} - v_{\mathrm{Pnp1}};$$

$$\frac{d[\mathrm{HYP}]}{dt} = +v_{\mathrm{Pnp1}} - v_{\mathrm{Hpt1}};$$

$$\frac{d[\mathrm{NAD}]}{dt} = +v_{\mathrm{G3PDH}} - v_{\mathrm{GAPDH}} + v_{\mathrm{ADH}} + v_{\mathrm{mitoNADH}};$$

$$\frac{d[\mathrm{NADH}]}{dt} = -v_{\mathrm{G3PDH}} + v_{\mathrm{GAPDH}} - v_{\mathrm{ADH}} - v_{\mathrm{mitoNADH}};$$

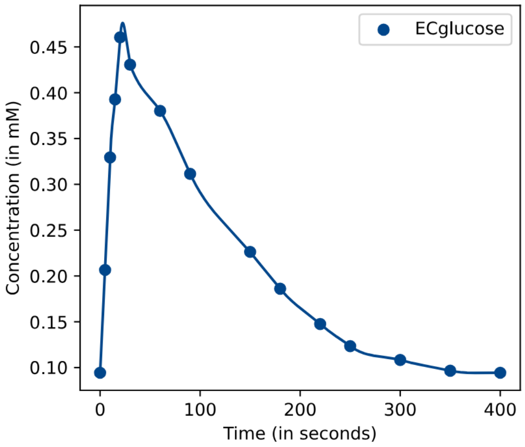

Fig. B: Cubic spline interpolated extracellular glucose.

6.2. Modeling feast/famine data

Data was retrieved from a feast/famine experiment [20]. The setup was feast/famine experimental setup was the following; the first phase was not sampled and was a steady state chemostat phase. Then, the feast/famine phase starts, this consists of a 20s feeding phase, where 43 mL of fresh medium was added followed by a 380 second without feed. This was repeated three times, resulting in three datasets.

6.2.1. Interpolation of extracellular glucose

Extracellular glucose was interpolated using cubic spline interpolation [2]. This was used as the stimulus input to the rest of the glycolysis model (Fig.).

7. Fitting multiple datasets simultaneously

Gradient descent based methods can be easily generalized to fit multiple datasets simultaneously by averaging gradients between datasets. This is similar in approach to many federated learning algorithms, where gradient averaging is common [21].

Figure C shows the loss landscape when fitting multiple datasets simultaneously compared to fitting individual datasets. While for the feast/famine glucose pulse datasets one and three we do not observe a better loss, we do observe a better loss for dataset two. Furthermore, by training on multiple datasets, the generalization capabilities of a kinetic model is expected to increase.

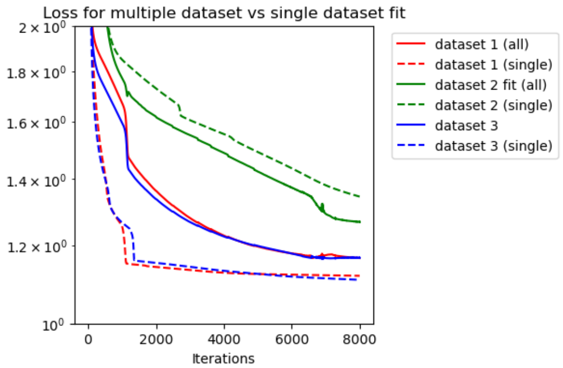

Fig. C: Loss landscape when fitting all datasets simultaneously versus individual dataset fitting. The dashed lines are models trained on individual feast/famine cycles, while the other lines are the fits of the individual dataset when fitting all datasets simultaneously.

8. Small subset of parameters explain dynamic behavior in systems biology models

As it was established that kinetic models could be trained using techniques from the Neural ODE field, we aimed to explain parameter importance during training, as it has been reported that systems biology models are often sloppy [22]. Sloppiness refers to a characteristic in models with many parameters, where only a few stiff parameter combinations influence the output time-series, while the output to other *sloppy* parameters is largely insensitive [23]. These sloppy parameters are thought to complicate the training process due to their insensitivity to the loss function, but also give an indication about the parameters that matter for the observed dynamic behavior of the system.

One of the SBML models that were retrieved from a previous collection of benchmark model was used for further analysis [24, 25]. The model describes the dynamic behavior of the epoR system, which is a widely studied pathway. We start the analysis by computing the cosine distance (see *Methods*) between the true parameter set from the SBML model and the initialized parameter set as well as the trained parameters (Fig. DA). There seems to be no clear difference in the average distance before and after training, although a small increase in density is observed closer to the optimum for the trained parameters. However, when performing Principal Component Analysis on the parameters (see *Methods*), a clear separation is observed between the parameter before and after training (Fig. DB). When looking at the loadings of the PCA, we observe that for the first principal component a few parameters are of

high importance for this separation (Fig. DC). Indeed, when we take only the first three parameters and redo the cosine distance plot, we observe that these parameters are very close to the true optimum, suggesting that only a subset of parameters are important to be precisely set (Fig. DD). This is in the literature known as *sloppy* models and has been suggested to be a general feature in systems biology models, either through the way they are constructed, or due to the actual underlying biological properties of robustness to parameters [25, 23]. We observe the same behavior for other SBML models, suggesting that this is a general feature of this type of systems. Furthermore, we observe that the parameters are typically distributed in log-space or even on an exponential distribution. Altogether, these results indicate that the ensemble of parameters can be informative for checking the respective importance of parameters in the model.

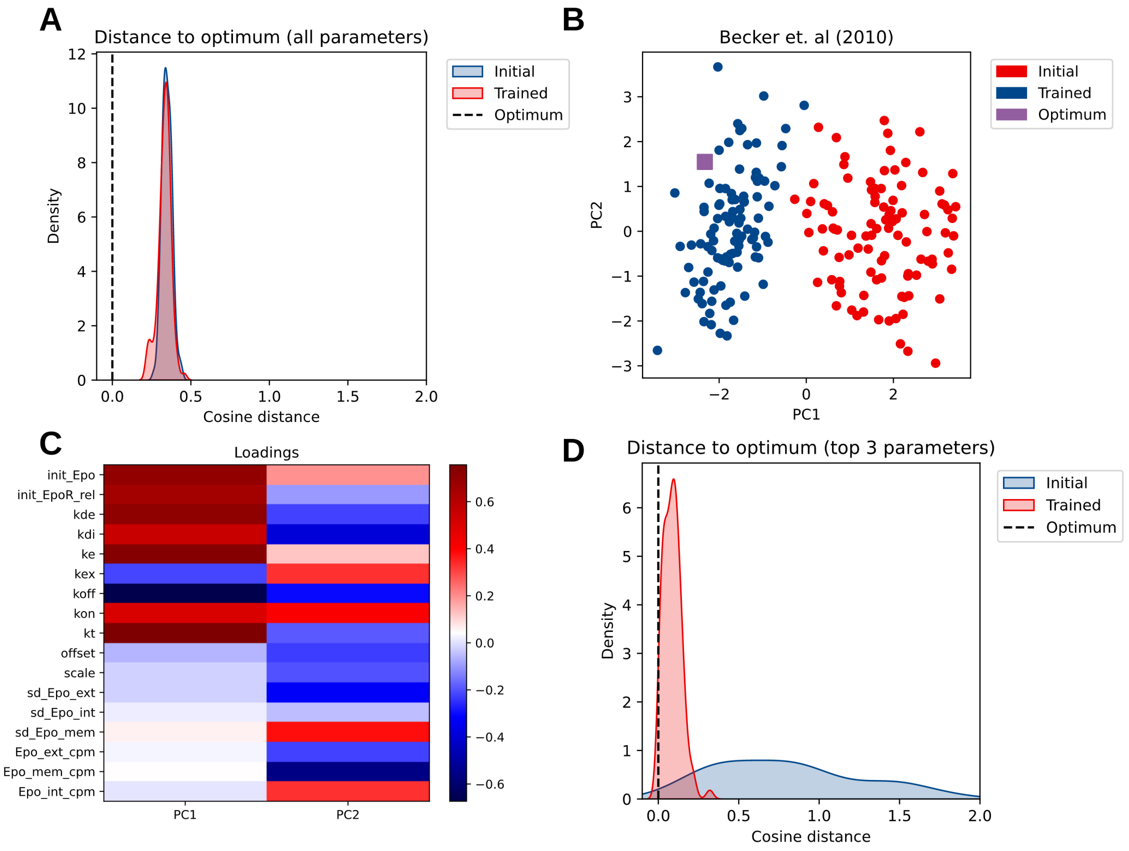

Fig. D: **Only few parameters are precisely set during training.** A) The cosine distance of the initialization parameter set and trained parameter set are compared to the true optimum parameter set. B) Principal Component Analysis of the initialization parameter set and parameters after training. The trained parameter lie closer in space to the optimum, although much variation exists. C) Loadings plot of the first two Principal Components reveal some parameters with large impact on covariance. D) Cosine distance for the top three parameters with the highest loadings. The distance is closer to the optimum, indicating a precise setting of some parameters.

Principal component analysis plots of parameter space

To show that the dynamic behavior observed in figure D, we show the behavior of the parameters before and after training for a few other models (Fig. E).

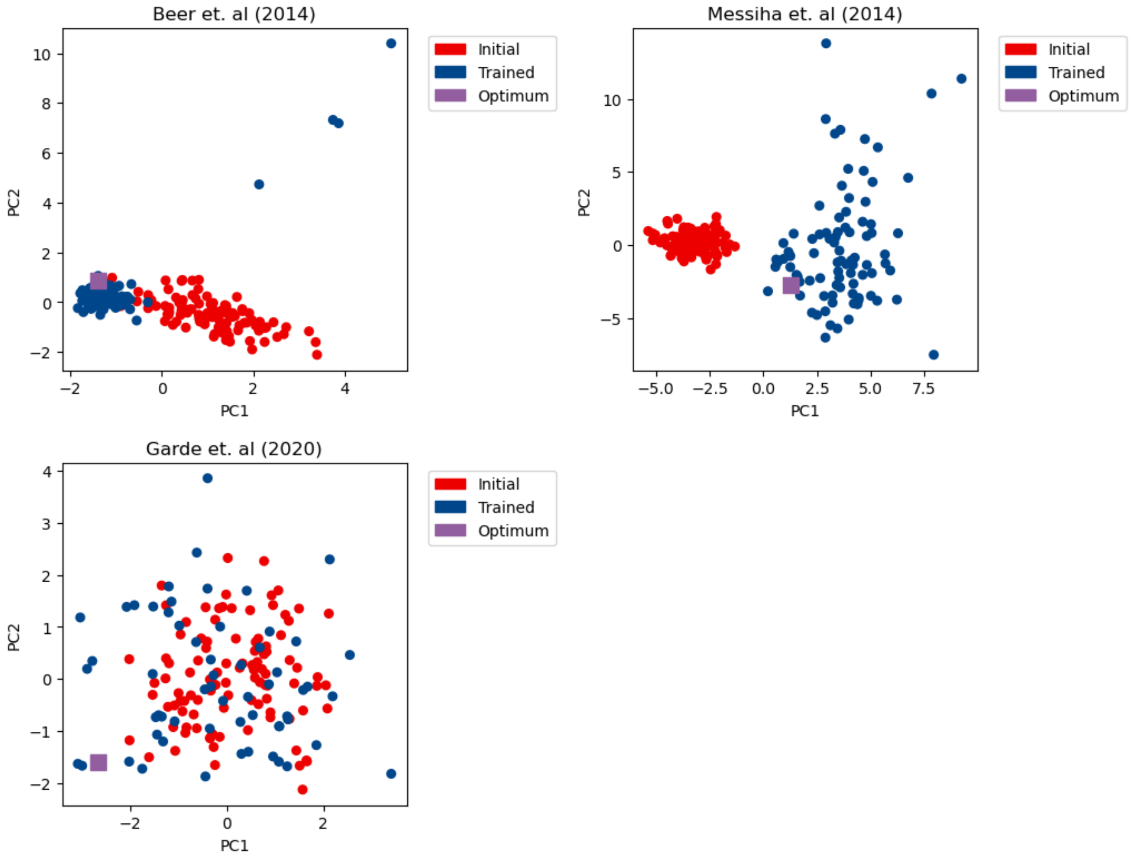

Fig. E: **Principal Component Analysis before and after training**
The three plots are the same models as figure 2D,E,F. As can be seen for the models that were properly trained, the region in parameter space after training for the first two plots is closer to the optimum than the poorly trained oscillating data.

9. An example of hybrid modeling

As shown in the main results, we show an example of how *jaxkineticmodel* can be easily combined with neural networks due to the flexibility of automatic differentiation in *JAX/Diffrax*. Here, we report the time-series for all masked reactions of the Garde model [31]. These results showcase how neural networks can be combined with mechanistic decriptions to model biological systems.

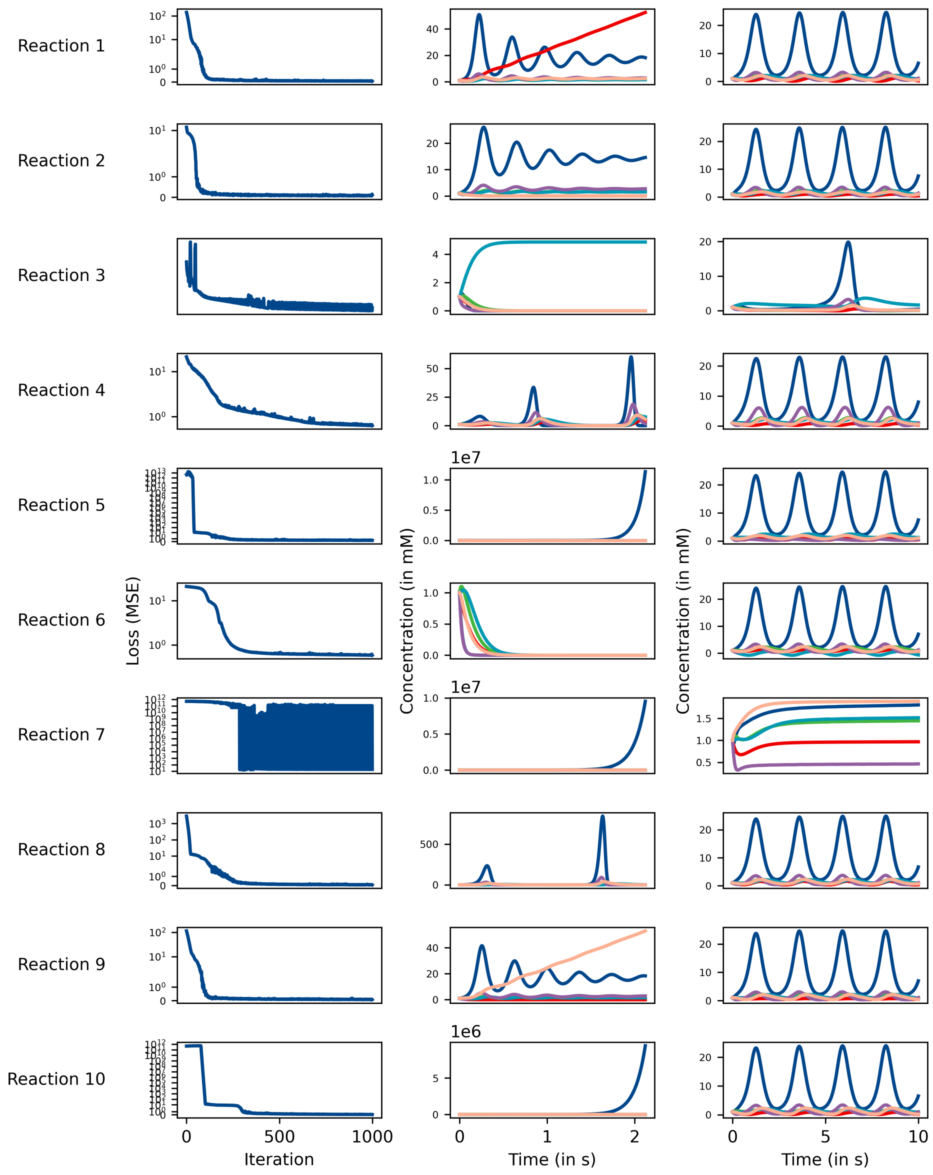

Fig. F: Time-series plots and loss during training for all masked examples. The left plot is the loss during training (1000 time-steps), the middle plot is the dynamics with a removed reaction, and the third plot is the dynamics after training. For almost all reactions, the true dynamics can be recovered, with the exception of reaction 3 and 7.

References

[1] R. T. Q. Chen, Y. Rubanova, J. Bettencourt, D. K. Duvenaud, Neural Ordinary Differential Equations, Advances in Neural Information Processing Systems 31

(2018).

[2] P. Kidger, On Neural Differential Equations, PhD thesis (2022).

[3] R. Mester, A. Landeros, C. Rackauckas, K. Lange, Differential methods for assessing sensitivity in biological models, PLoS Computational Biology 18 (6) (2022). doi:10.1371/journal.pcbi.1009598.

[4] R. Pascanu, T. Mikolov, Y. Bengio, On the difficulty of training recurrent neural networks, in: 30th International Conference on Machine Learning, ICML 2013, no. PART 3, 2013.

[5] J. Bradbury, R. Frostig, P. Hawkins, M. J. Johnson, C. Leary, D. Maclaurin, G. Necula, A. Paszke, J. VanderPlas, S. Wanderman-Milne, et al., Jax: composable transformations of python+ numpy programs, https://jax.readthedocs.io/en/latest/ (2018).

[6] D. Lao-Martil, J. P. Schmitz, B. Teusink, N. A. van Riel, Elucidating yeast glycolytic dynamics at steady state growth and glucose pulses through kinetic metabolic modeling, Metabolic Engineering 77 (2023). doi:10.1016/j.ymben.2023.03.005.

[7] S. Gopalakrishnan, S. Dash, C. Maranas, K-fit: An accelerated kinetic parameterization algorithm using steady-state fluxomic data, Metabolic engineering 61 (2020) 197–205.

[8] M. Hu, P. F. Suthers, C. D. Maranas, Ketchup: Parameterizing of large-scale kinetic models using multiple datasets with different reference states, Metabolic engineering 82 (2024) 123–133.

[9] L. Miskovic, V. Hatzimanikatis, Production of biofuels and biochemicals: In need of an ORACLE, Trends in Biotechnology 28 (8) (2010) 391–397. doi:10.1016/j.tibtech.2010.05.003.

[10] S. Choudhury, M. Moret, P. Salvy, D. Weilandt, V. Hatzimanikatis, L. Miskovic, Reconstructing Kinetic Models for Dynamical Studies of Metabolism using Generative Adversarial Networks, Nature Machine Intelligence 2022 4:8 4 (8) (2022) 710–719. doi:10.1038/s42256-022-00519-y.

[11] S. Choudhury, B. Narayanan, M. Moret, V. Hatzimanikatis, L. Miskovic, Generative machine learning produces kinetic models that accurately characterize intracellular metabolic states, Nature Catalysis 7 (10) (2024) 1086–1098.

[12] T. Groves, N. L. Cowie, L. K. Nielsen, Bayesian regression facilitates quantitative modeling of cell metabolism, ACS Synthetic Biology 13 (4) (2024) 1205–1214.

[13] F. Fröhlich, D. Weindl, Y. Schälte, D. Pathirana, Ł. Paszkowski, G. T. Lines, P. Stapor, J. Hasenauer, Amici: high-performance sensitivity analysis for large ordinary differential equation models, Bioinformatics 37 (20) (2021) 3676–3677.

[14] Y. Schälte, F. Fröhlich, P. J. Jost, J. Vanhoefer, D. Pathirana, P. Stapor, P. Lakrisenko, D. Wang, E. Raimúndez, S. Merkt, L. Schmiester, P. Städter, S. Grein, E. Dudkin, D. Doresic, D. Weindl, J. Hasenauer, pyPESTO: a modular and scalable tool for parameter estimation for dynamic models, Bioinformatics 39 (11) (2023). doi:10.1093/bioinformatics/btad711.

[15] L. Contento, P. Stapor, D. Weindl, J. Hasenauer, A more expressive spline representation for sbml models improves code generation performance in amici, in: International Conference on Computational Methods in Systems Biology, Springer, 2023, pp. 36–43.

[16] A. C. Hindmarsh, P. N. Brown, K. E. Grant, S. L. Lee, R. Serban, D. E. Shumaker, C. S. Woodward, Sundials: Suite of nonlinear and differential/algebraic equation solvers, ACM Transactions on Mathematical Software (TOMS) 31 (3) (2005) 363–396.

[17] J. R. Karr, A. H. Williams, J. D. Zucker, A. Raue, B. Steiert, J. Timmer, C. Kreutz, D. P. E. C. Consortium, S. Wilkinson, B. A. Allgood, et al., Summary of the dream8 parameter estimation challenge: toward parameter identification for whole-cell models, PLoS computational biology 11 (5) (2015) e1004096.

[18] M. Hucka, L. Smith, F. Bergmann, S. Keating, Sbml test suite release 3.3. 0, Zenodo (2017).

[19] C. Welsh, J. Xu, L. Smith, M. König, K. Choi, H. M. Sauro, libroadrunner 2.0: a high performance sbml simulation and analysis library, Bioinformatics 39 (1) (2023) btac770.

[20] C. Suarez-Mendez, A. Sousa, J. Heijnen, A. Wahl, Fast “Feast/Famine” Cycles for Studying Microbial Physiology Under Dynamic Conditions: A Case Study with Saccharomyces cerevisiae, Metabolites 4 (2) (2014). doi:10.3390/metabo4020347.

[21] B. McMahan, E. Moore, D. Ramage, S. Hampson, B. A. y Arcas, Communication-efficient learning of deep networks from decentralized data, in: Artificial intelligence and statistics, PMLR, 2017, pp. 1273–1282.

[22] R. N. Gutenkunst, J. J. Waterfall, F. P. Casey, K. S. Brown, C. R. Myers, J. P. Sethna, Universally Sloppy Parameter Sensitivities in Systems Biology Models, PLOS Computational Biology 3 (10) (2007) e189. `doi:10.1371/JOURNAL.PCBI.0030189`.
URL `https://journals.plos.org/ploscompbiol/article?id=10.1371/journal.pcbi.0030189`

[23] M. K. Transtrum, B. B. MacHta, J. P. Sethna, Geometry of nonlinear least squares with applications to sloppy models and optimization, Physical Review E - Statistical, Nonlinear, and Soft Matter Physics 83 (3) (2011) 036701. `doi:10.1103/PHYSREVE.83.036701/FIGURES/29/MEDIUM`.

[24] V. Becker, M. Schilling, J. Bachmann, U. Baumann, A. Raue, T. Maiwald, J. Timmer, U. Klingmüller, Covering a broad dynamic range: Information processing at the erythropoietin receptor, Science 328 (5984) (2010). `doi:10.1126/science.1184913`.

[25] H. Hass, C. Loos, E. Raimúndez-Álvarez, J. Timmer, J. Hasenauer, C. Kreutz, Benchmark problems for dynamic modeling of intracellular processes, Bioinformatics 35 (17) (2019). `doi:10.1093/bioinformatics/btz020`.

[26] Lakrisenko, P., Pathirana, D., Weindl, D. & Hasenauer, J. Benchmarking methods for computing local sensitivities in ordinary differential equation models at dynamic and steady states. *Plos One*. **19**, e0312148 (2024)

[27] Philipps M, Körner A, Vanhoefer J, Pathirana D, Hasenauer J. Non-Negative Universal Differential Equations With Applications in Systems Biology. arXiv preprint arXiv:240614246. 2024;.

[28] Noordijk B, Garcia Gomez ML, ten Tusscher KHWJ, de Ridder D, van Dijk ADJ, Smith RW. The rise of scientific machine learning: a perspective on combining mechanistic modelling with machine learning for systems biology. Frontiers in Systems Biology. 2024;4:1407994. doi:10.3389/FSYSB.2024.1407994/BIBTEX.

[29] Rackauckas C, Ma Y, Martensen J, Warner C, Zubov K, Supekar R, et al. Universal differential equations for scientific machine learning. arXiv preprint arXiv:200104385. 2020;.

[30] Etcheverry, M., Levin, M., Moulin-Frier, C. & Oudeyer, P. SBMLtoODEjax: Efficient Simulation and Optimization of Biological Network Models in JAX. *ArXiv Preprint ArXiv:2307.08452*. (2023)

[31] Garde R, Ibrahim B, Schuster S. Extending the minimal model of metabolic oscillations in Bacillus subtilis biofilms. Scientific Reports 2020 10:1. 2020;10(1):1–11. doi:10.1038/s41598-020-62526-6.
